# Rape Yield Estimation Considering Non-Foliar Green Organs Based on the General Crop Growth Model

**DOI:** 10.34133/plantphenomics.0253

**Published:** 2024-09-17

**Authors:** Shiwei Ruan, Hong Cao, Shangrong Wu, Yujing Ma, Wenjuan Li, Yong Jin, Hui Deng, Guipeng Chen, Wenbin Wu, Peng Yang

**Affiliations:** ^1^ School of Information and Communication Engineering, North University of China, Taiyuan 030051, China.; ^2^ State Key Laboratory of Efficient Utilization of Arid and Semi-arid Arable Land in Northern China (the Institute of Agricultural Resources and Regional Planning), Chinese Academy of Agricultural Sciences, Beijing 100081, China.; ^3^ Key Laboratory of Agricultural Remote Sensing, Ministry of Agriculture and Rural Affairs/Institute of Agricultural Resources and Regional Planning, Chinese Academy of Agricultural Sciences, Beijing 100081, China.; ^4^ Institute of Agricultural Economics and Information, Jiangxi Academy of Agricultural Sciences, Nanchang 330200, China.

## Abstract

To address the underestimation of rape yield by traditional gramineous crop yield simulation methods based on crop models, this study used the WOFOST crop model to estimate rape yield in the main producing areas of southern Hunan based on 2 years of field-measured data, with consideration given to the photosynthesis of siliques, which are non-foliar green organs. First, the total photosynthetic area index (TPAI), which considers the photosynthesis of siliques, was proposed as a substitute for the leaf area index (LAI) as the calibration variable in the model. Two parameter calibration methods were subsequently proposed, both of which consider photosynthesis by siliques: the TPAI-SPA method, which is based on the TPAI coupled with a specific pod area, and the TPAI-Curve method, which is based on the TPAI and curve fitting. Finally, the 2 proposed parameter calibration methods were validated via 2 years of observed rape data. The results indicate that compared with traditional LAI-based crop model calibration methods, the TPAI-SPA and TPAI-Curve methods can improve the accuracy of rape yield estimation. The estimation accuracy (*R*^2^) for the total weight of storage organs (TWSO) and above-ground biomass (TAGP) increased by 9.68% and 49.86%, respectively, for the TPAI-SPA method and by 14.04% and 42.94%, respectively, for the TPAI-Curve method. Thus, the 2 calibration methods proposed in this study are of important practical importance for improving the accuracy of rape yield simulations. This study provides a novel technical approach for utilizing crop growth models in the yield estimation of oilseed crops.

## Introduction

Rape is the third largest oilseed crop in the world, with an annual production of approximately 2.51 million tons (Food and Agriculture Organization of the United Nations; https://www.fao.org/faostat), accounting for 16% of global major oil and fat production. Rape is rich in nutrients such as oil and protein [1] and is a crucial raw material for edible oil, feed protein, and biodiesel production. It holds important importance in the modern food, industry, and bioenergy sectors and in ecological conservation [2]. Therefore, dynamic, accurate, and large-scale growth simulations and yield predictions for rape are crucial.

The accumulation of dry matter in crops such as wheat, maize, and rice mainly involves photosynthesis performed by plant leaves, and growth is monitored mainly by considering the canopy leaf area index (LAI) [3–7]. The LAI represents the proportion of leaf area to land area per unit. For crops in which leaves serve as the primary photosynthetic organs, the LAI serves as a fundamental parameter in the structural makeup of the canopy, directly influencing light interception and photosynthetic productivity and thereby determining crop growth status and yield formation. Hence, the LAI is a critical parameter for achieving crop growth simulations and yield estimations [8]. Furthermore, research has revealed a close correlation between the LAI and yield at specific growth stages in wheat [9–12]. However, leaves are not the only organs in crops that can photosynthesize. The photosynthesis of non-foliar green organs such as siliques, pods, stems, and petioles contributes to yield [13]. Rape exhibits distinctive photosynthetic organ succession characteristics, and its siliques, which are the most active non-foliar green organs, play a crucial role in yield formation [14]. During the growth and development of rape, the primary photosynthetic organs shift gradually from the leaves to the siliques [15]. Leaves are the primary photosynthetic organs from the seedling stage to the flowering period. After flowering, the siliques continue to grow, and photosynthesis begins to occur in the silique walls. Both the leaves and silique walls participate in photosynthesis [16]. As the siliques mature, the leaves gradually senesce, and the plants rely primarily on photosynthesis in the silique wall for the grain-filling stage. The maximum surface area of rape siliques is 1.54 times greater than the maximum leaf area, and the net photosynthetic rate, transpiration rate, and light radiation intensity in rape siliques are greater than those in rape leaves [17]. Therefore, the simulation of rape yield needs to consider the leaf area and silique surface area.

Crop growth models developed on the basis of agronomic knowledge and computer technology have important advantages in monitoring crop growth. They contribute to providing timely and accurate crop yield assessments and macro decision support for government agencies [18]. In the 1960s, scholars in the Netherlands and the United States pioneered research on crop models. Since then, crop growth models have rapidly evolved. By comprehensively analyzing meteorological, crop, soil, and field management factors, these models simulate processes such as crop photosynthesis, respiration, transpiration, and nutrient allocation. These methods simulate crop canopy and soil variables at any given time, ultimately leading to highly accurate estimations of crop yields [19,20]. With the advancement of crop growth simulation technology, crop models have evolved into 2 main branches: general crop models and specialized crop models. Specialized crop models establish independent parameter models for different crop varieties. Widely applied examples include the decision support system for agrotechnology transfer (DSSAT) and agricultural production systems simulator (APSIM) series models, which have been successfully employed in the growth and yield simulation of various crops, such as maize [21], wheat [22], and soybean [23]. Although specialized crop models offer high accuracy in crop growth and yield simulations, the somewhat independent modules among these models [24] result in poor parameter generalizability. This makes it challenging to calibrate these models for different crop types and study areas. In contrast to specialized crop models, general crop models can simulate the growth of different crops via the same input parameters [25]. Unified input and output parameters importantly reduce the time required for model calibration. General crop models also exhibit strong data transferability [26], facilitating the integration of large models and aligning with the trend toward the scalability and integration of crop growth models. The world food studies (WOFOST) crop model is a typical representative of general crop models. By adjusting external input parameters such as crop, soil, and field management, processes such as crop transpiration, respiration, photoassimilate allocation, and biomass formation can be performed. This allows precise control over the crop growth process [27]. In recent years, many scholars have conducted crop yield simulations under different conditions via the WOFOST crop model. This model has been widely applied to various crops, including wheat [20,28–31], maize [5,6,10,32–34], rice [3,7,35], goji berries [36], vegetables [37,38], and fruit trees [4,39]. However, the use of the WOFOST crop model in the growth simulation of oilseed crops such as soybean and rape has been relatively limited, leading to the underestimation of yields.

To address the underestimation of rape yields by traditional gramineous crop yield simulation methods based on crop models, this study proposed the total photosynthetic area index (TPAI), adding the photosynthesis parameter by non-foliar green organs (i.e., siliques) as the calibration variable in the WOFOST crop model for yield estimation. Furthermore, 2 parameter calibration methods, namely, methods based on the TPAI coupled with specific pod area (TPAI-SPA) and the TPAI coupled with curve fitting (TPAI-Curve), were proposed to increase the simulation accuracy of rape yield via the WOFOST model. Finally, utilizing 2 years of field measurement data from the main rape-producing region in southern Hunan, China, an accuracy assessment of rape yield estimation based on the WOFOST crop model is conducted.

## Materials and Methods

### Materials

#### Study area

**Fig. 1.**
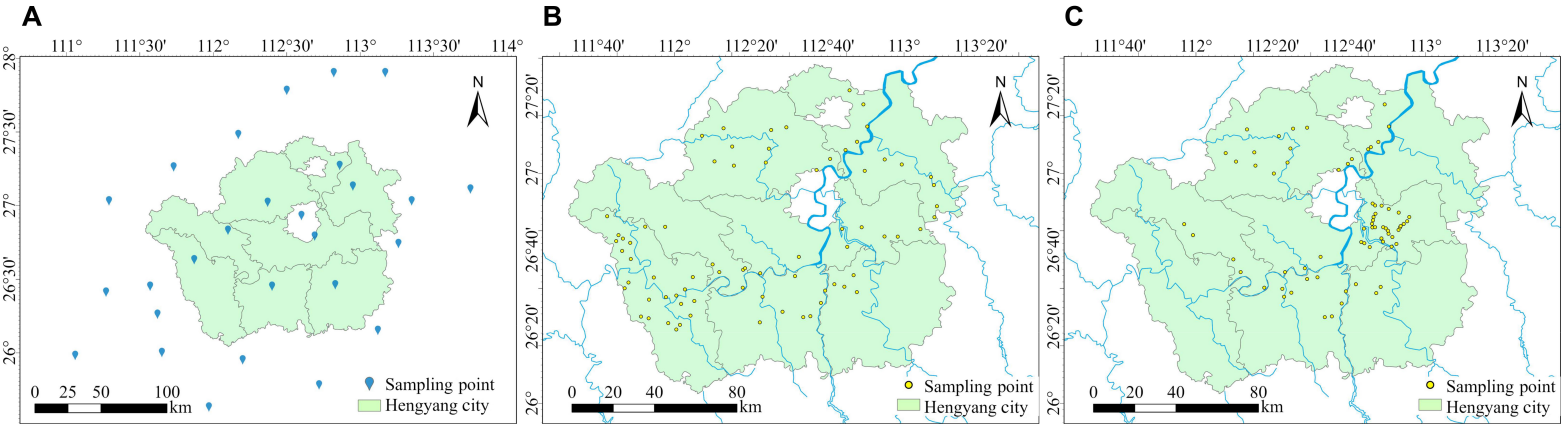
Study area. (A) Locations of weather stations in Hengyang city and surrounding counties. (B) Ground sampling point distribution in 2020–2021. (C) Ground sampling point distribution in 2022–2023.

The study area is located in Hengyang city, southern Hunan, China. Hunan Province is located in the middle and lower reaches of the Yangtze River and is one of the main regions for rape cultivation in China. According to the 2022/2023 data from the Hunan Provincial Bureau of Statistics (https://tjj.hunan.gov.cn/), the rape sowing area in Hengyang city is approximately 200,000 ha, accounting for more than 90% of the total sowing area of oilseed crops in the region, with an annual yield of approximately 320,000 tons. The study area is located in the central-southern part of Hunan Province, with coordinates ranging from approximately 110°32′16″E to 113°16′32″E longitude and 26°7′5″N to 27°28′24″N latitude. The elevation is 81 m, and the predominant soil type is red soil. The average temperature during the rape growing season is 14.8 °C, with 3.3 h of sunlight per day and a total precipitation of 493 mm. The cropping system in the study area follows a water–drought rotation pattern, with winter rape followed by summer rice cultivation. The study area is illustrated in Fig. 1.

#### Meteorological data

The WOFOST crop model is driven by meteorological data to simulate daily crop yields. The meteorological data used in this study include data from 27 meteorological stations in Hengyang city and its surrounding counties and cities (http://hn.cma.gov.cn/), as shown in Fig. 1A. The meteorological data collected during the rape growth period in the study area are presented in Table 1.

**Table 1.** Meteorological data in the study area

Year	County	Longitude	Latitude	Average daily minimum temperature/°C	Average daily maximum temperature/°C	Average daily precipitation/mm	Average daily vapor pressure/kPa	Average daily wind speed/m·s^−1^	Average daily sunshine duration/h
2020-2021	Changning	112.40	26.42	10.24	17.20	2.84	1.60	1.54	3.06
	Hengdong	112.95	27.10	9.98	17.09	2.50	1.57	1.39	3.75
	Hengnan	112.68	26.75	9.92	17.37	2.36	1.62	2.18	3.32
	Hengshan	112.87	27.23	9.70	16.81	1.98	1.62	3.84	3.31
	Hengyang	112.37	26.98	9.87	16.89	1.92	1.60	2.33	3.08
	Leiyang	112.83	26.43	10.21	17.12	2.06	1.64	1.82	3.18
	Qidong	112.10	26.80	10.26	16.92	2.27	1.60	2.16	2.75
	Qiyang	111.87	26.60	10.77	17.72	2.18	1.60	1.26	3.43
2022-2023	Changning	112.40	26.42	10.54	17.61	1.99	1.43	1.68	3.55
	Hengdong	112.95	27.10	9.25	17.42	2.29	1.47	2.11	3.68
	Hengnan	112.68	26.75	9.96	17.65	1.95	1.44	2.40	3.75
	Hengshan	112.87	27.23	9.54	16.97	1.94	1.40	3.97	3.69
	Hengyang	112.37	26.98	9.96	17.45	1.94	1.41	2.28	3.61
	Leiyang	112.83	26.43	10.36	17.42	2.27	1.40	1.88	3.50
	Qidong	112.10	26.80	10.03	17.35	2.04	1.32	1.92	3.69
	Qiyang	111.87	26.60	10.45	17.76	1.78	1.41	1.82	3.52

#### Field measurement data

The field measurement data included 2 years of data from 2020 to 2021 and 2022 to 2023. Each year, data collection is conducted according to the rape phenology in the research area. Five key rape growth stages were selected for field measurement and sampling, and the measurement times and data are shown in Table 2. In the 2020–2021 period, a total of 77 rape sampling points were arranged, as shown in Fig. 1B; in the 2022–2023 period, a total of 70 rape sampling points were arranged, as shown in Fig. 1C. In the 2020–2021 period, the sampling points were randomly distributed along the Xiangjiang main rape production area. In the 2022–2023 period, a combined centralized and distributed arrangement was used for rape sampling points, aimed at analyzing the spatial adaptability of rape yield simulations. Given that the meteorological station points in various counties and cities do not precisely correspond to rape sampling points, this study employed the inverse distance weighting (IDW) algorithm for spatial interpolation of meteorological data to obtain the corresponding meteorological data for the rape sampling points.

**Table 2.** Field experiment times and measured data

No.	Rape phenology	2020–2021	2022–2023	Measured data
1	Seedling stage	01.10–01.16	01.11–01.15	Crop data: dry weight, wet weight, length, width and thickness of leaves, stems, siliques, and flowers, plant height; LAI; soil data: moisture content
2	Budding stage	02.21–02.28	02.06–02.12	
3	Flowering stage	03.14–03.20	03.01–03.06	
4	Milky stage	04.03–04.16	03.26–04.01	
5	Maturity stage	04.29–05.03	04.18–04.21	

Notes: For the first additional measurement of plant row spacing, for the fifth additional measurement of thousand-grain weight and number of seeds per thousand siliques.

The field measurement data mainly included crop, soil, and field management parameters. For crop parameters, calipers were used to measure the length, width, and thickness of rape leaves, as well as the length and diameter of siliques; a steel ruler was used to measure the spacing of rape rows. The plant and silique counts were conducted on unit area quadrats, and the corresponding densities were calculated via Eq. 1:

$$\rho =\frac{10^{4}\cdot n_{a}}{100^{2}}$$ (1)

where *n_a_* is the number of rape plants or siliques within the quadrat and *ρ* is the density of plants or siliques measured in individuals per m^2^.

The LAI was measured via an LAI-2200 canopy analyzer. Three measurements were taken during the periods of 06:30 to 09:00 or 14:30 to 19:00 to avoid direct sunlight, and the average was calculated. After the plants were dissected into their components, the wet weights of the leaves, stems, and siliques were measured via a precision balance. The leaves, stems, and siliques were subjected to 105 °C for 2 h to stop metabolic activity, followed by drying at a constant temperature of 80 °C for 48 h until a constant weight was achieved. The dry weight of each part was then measured, and the plant water content was calculated via Eq. 2:

$$\theta =1-\frac{m_{d}}{m_{w}}$$ (2)

where *θ* represents the plant water content, *m_d_* represents the dry weight, and *m_w_* represents the wet weight.

The rape yield was obtained by measuring the thousand-seed weight within a mature unit plot and was calculated via Eq. 3:

$$N=\frac{m_{b}}{n_{b}}\times 1000$$ (3)

where *N* is the thousand-seed weight, measured in g; *m_b_* is the yield per unit plot, measured in g; and *n_b_* is the number of rapes per unit plot.

The soil moisture content was measured via time domain reflectometer (TDR) soil moisture sensors, with 5 measurement points established within a 5-m radius centered on each sampling point, and the soil moisture content was measured at depths of 5 and 25 cm.

### Methods

#### Calculation of the TPAI

Rape plants exhibit distinctive morphological and photosynthetic organ succession characteristics. As rape grows and develops, its photosynthetic organs gradually transition from leaves to siliques [15]. Therefore, in simulating the growth and yield of rape, it is essential to fully consider the photosynthesis of siliques in the later stages of rape growth and their contribution to yield. To model the growth and yield formation processes of rape more accurately, we propose the TPAI, which considers silique photosynthesis to improve the simulation accuracy of rape and uses it as a calibration variable in the calibration process of the crop growth model, replacing the traditional LAI.

The silique of rape has a 3-dimensional structure; thus, it is difficult to measure the silique area index (SAI) via portable instruments. Additionally, our research area is large; it spans 150 km from east to west and is located in hilly mountainous regions. Thus, it is challenging to determine the LAI and SAI of rape via agronomic methods. Furthermore, we compared 2 years of measured rape data and found that the canopy leaf area and LAI of rape were highly correlated. Therefore, we propose a method to infer the SAI on the basis of measurements of the LAI via portable instruments.

**Fig. 2.**
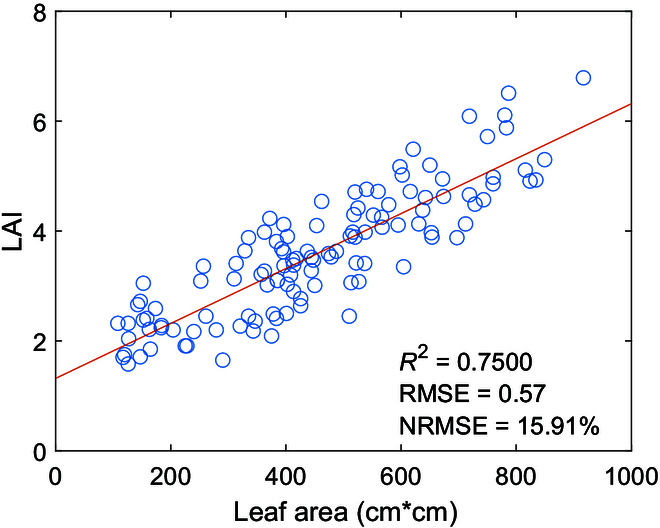
Relationship between single leaf area and LAI. A total of 117 data points were selected for plotting, which included all the sampling points during the rape milky period, as well as some points during the rape flowering and maturity periods.

There was a important linear relationship between the estimated leaf area and LAI measured by the LAI-2200, as shown in Fig. 2, with a regression accuracy metric determination coefficient (*R*^2^), root mean square error (RMSE), and normalized root mean square error (NRMSE) of 0.75%, 0.57%, and 15.91%, respectively. On the basis of this relationship, we calculated the TPAI, and the calculation method for the TPAI was as follows:

First, the regression relationship between the estimated leaf area and LAI was measured by the LAI-2200 equation (i.e., the red regression line in Fig. 2), as shown in Eq. 4:

$$\mathrm{LAI}=0.005\cdot S_{L}+1.318$$ (4)

In the equation, *S_L_* is the estimated leaf area of rape. The shape of rape leaves can be equated to an ellipse, and the area of rape leaves can be calculated by measuring their length and width based on the formula for the area of an ellipse.

Then, based on the above regression relationship, the relationship between the estimated silique surface area and the SAI was calculated. Specifically, the estimated silique surface area was substituted to calculate the SAI and the TPAI as follows:

$$\mathrm{SAI}=0.005\cdot S_{P}+1.318$$ (5)

TPAI=LAI+SAI (6)

where *S_P_* is the estimated silique surface area of the rape.

#### Rape parameter calibration method

Traditional parameter calibration methods for the WOFOST crop model neglect the photosynthesis process in rape siliques, resulting in a important underestimation of rape yield. Therefore, this study fully considered the TPAI and proposed 2 parameter calibration methods created specifically for rape yield estimation, namely, the TPAI-SPA and the TPAI-Curve, in the WOFOST crop model.

1. TPAI-SPA parameter calibration method

**Fig. 3.**
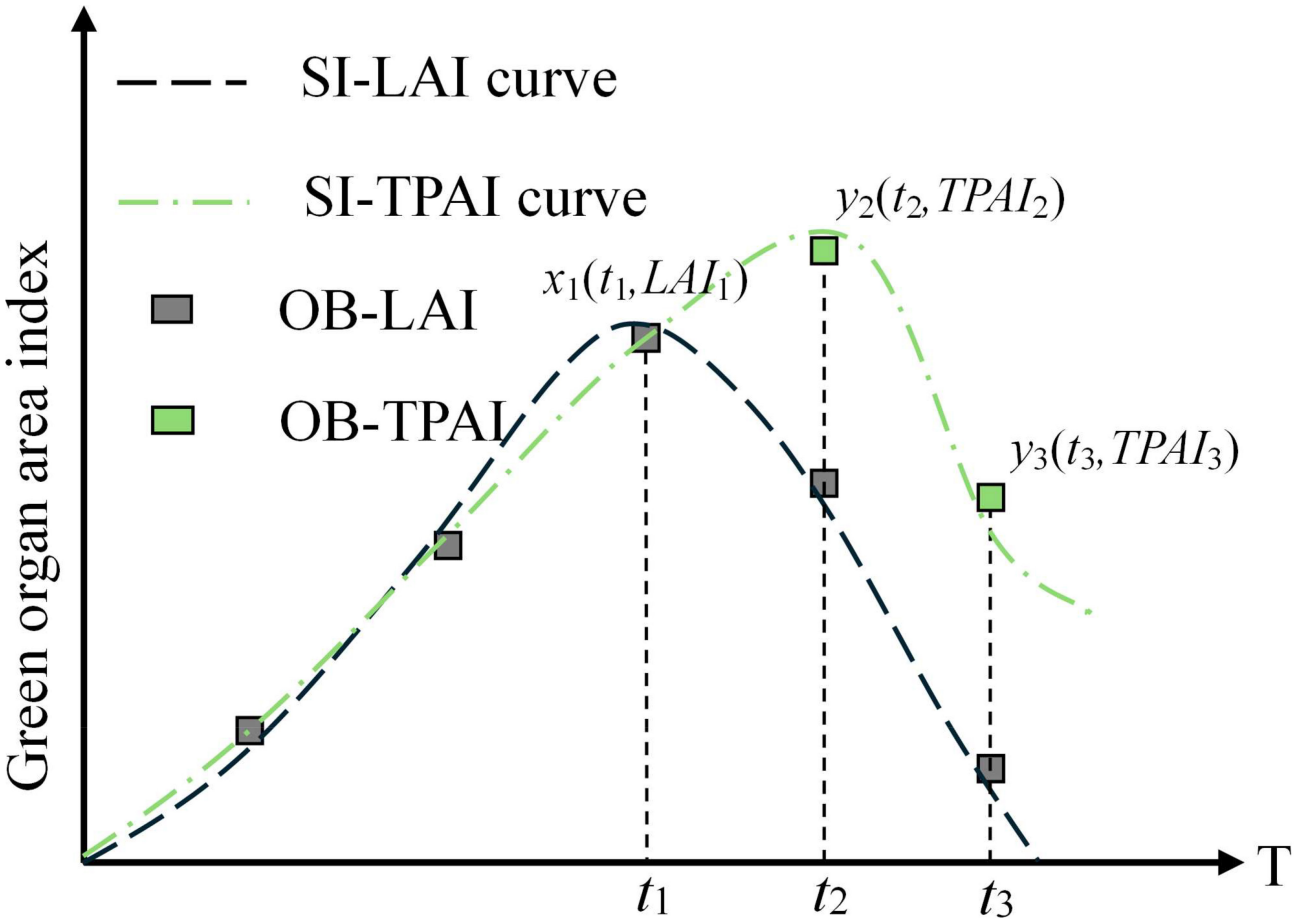
TPAI-SPA parameter calibration process. OB represents the observed values, and SI represents the simulated values. Notes: The rape siliques have not yet appeared in the first 3 periods of the measured time, the SAI is 0, and the TPAI is only equal to the LAI.

The TPAI-SPA parameter calibration method involves using TPAI values to calibrate the TPAI values output by the WOFOST crop model. By calibrating the WOFOST model parameters represented by SPA, the simulation of the TPAI curve in the crop model is adjusted to better match the observed TPAI values (calculated by the LAI and SAI via Eqs. 5 and 6). This process aims to achieve precise simulation of rape growth and yield formation. The TPAI-SPA parameter calibration process is illustrated in Fig. 3.

The steps of the TPAI-SPA calibration were as follows:

a. Adjust the field management parameters: The start and end dates of the model simulation were set on the basis of the actual sowing and harvesting times. The initial dry matter weight was adjusted on the basis of the actual sowing amount of rape. Parameters related to accumulated temperature and absorbed light energy were adjusted on the basis of the developmental period, and the TPAI was measured at various time points. The dry matter allocation coefficient, dry matter conversion efficiency, and CO_2_ assimilation-related parameters were adjusted on the basis of the yield at each time point. The crop model parameters were gradually adjusted, and the model was run. The simulated and observed results were compared, and adjustments were made to make the simulated TPAI curve consistent with the measured TPAI values at each growth stage.

b. The preset SPA for the crop parameters was 0.007. On the basis of whether the simulated TPAI curve reached the observed value, the SPA was adjusted in steps of 0.0005 to 0.001 to ensure that the simulated TPAI curve was closer to the observed TPAI value.

c. The crop model parameters were further adjusted to obtain a combination that aligned the simulated TPAI curve with the TPAI calibration curve, resulting in the calibrated crop model parameters.

2. TPAI-Curve parameter calibration method

**Fig. 4.**
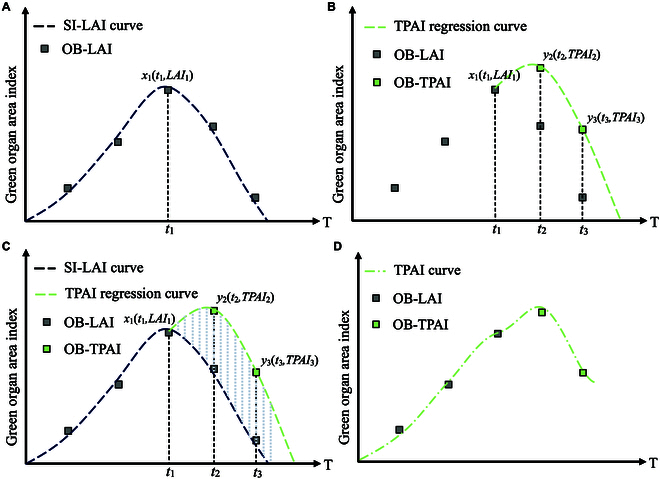
TPAI-Curve parameter calibration process. OB represents the observed values, and SI represents the simulated values. (A) Step 1. (B) Step 2. (C) Step 3. (D) Step 4 and Step 5. Notes: The rape siliques have not yet appeared in the first 3 periods of the measured time, the SAI is 0, and the TPAI is only equal to the LAI.

The TPAI-Curve parameter calibration method involves using a TPAI-fitted curve to calibrate the TPAI curve simulated by the WOFOST crop model. The parameter calibration process for the TPAI-Curve calibration method is illustrated in Fig. 4, and the fitting steps were as follows:

a. According to the first step of the TPAI-SPA parameter calibration method, the crop model parameters were adjusted such that the simulated LAI curve was consistent with the observed LAI values at each growth stage. The peak value of this LAI curve was obtained and is denoted as point *x*_1_(*t*_1_, *LAI*_1_).

b. The point *x*_2_(*t*_2_, *LAI*_2_) corresponding to the highest observed TPAI value after the *t*_1_ growth stage was found.

c. The TPAI before the measured production period was chosen as point *x*_3_(*t*_3_, *LAI*_3_).

d. A corresponding quadratic TPAI curve was fitted using points *x*_1_, *x*_2_, and *x*_3_. The points $\begin{array}{c} \{p_{1}\left(t_{1},{\text{TPAI}}_{1}^{s}\right),p_{2}\left(t_{2},{\text{TPAI}}_{2}^{s}\right),p_{3} \end{array}$$\begin{array}{c} \left(t_{3},{\text{TPAI}}_{3}^{s}\right),\cdots ,p_{n}\left(t_{n},{\text{TPAI}}_{n}^{s}\right)\} \end{array}$ corresponding to the dates were output as the TPAI calibration curve.

e. The crop model parameters were adjusted to identify a combination that aligned the simulated TPAI curve with the TPAI calibration curve, resulting in the calibrated crop model parameters.

#### Rape yield estimation process

**Fig. 5.**
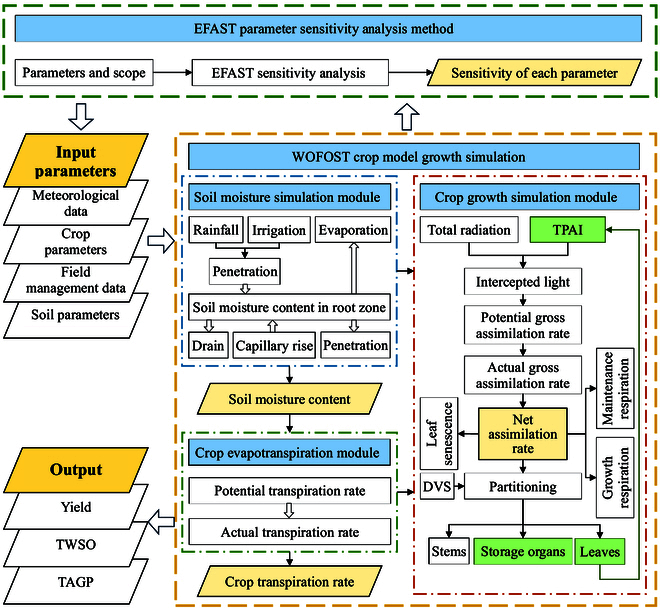
Overall technical roadmap.

The overall technical roadmap of this study is illustrated in Fig. 5. First, for parameter sensitivity analysis, the crop model parameters are set within a specified range. The extended Fourier amplitude sensitivity test (EFAST) parameter sensitivity analysis method is then employed to calculate the impact of model parameters on the accuracy of yield simulation in the WOFOST crop model. Second, meteorological data and crop, soil, and field management parameters are input into the crop model. Combined with the proposed TPAI-SPA and TPAI-Curve calibration methods, which consider the photosynthetic characteristics of rape siliques, the model simulates the growth, development, and yield formation process of rape. Finally, on the basis of the EFAST parameter sensitivity analysis results, continuous optimization and refinement of the model parameters are conducted to ensure that the simulated indicators of the crop model align with the field measurements.

Parameter sensitivity analysis is a crucial method for quantifying the impact of each parameter on crop growth indicators in crop models, aiding in the more accurate calibration of model parameters. In this study, the PCSE/WOFOST model developed in the Python environment employed the EFAST parameter sensitivity analysis method for analyzing the sensitivity of crop model parameters. The EFAST algorithm combines the advantages of the Sobol and FAST parameter sensitivity analysis methods, providing efficient handling of nonlinear global problems with multiple parameters [40]. For each sensitivity analysis iteration, the EFAST algorithm requires the algorithm to run *n* × *p* times, where *n* is the number of samples, *p* is the number of parameters, and the number of samples must exceed 65 times the number of parameters. In the experiment, a total of 59 model parameters were selected for sensitivity analysis, with 5,000 sampling iterations meeting the requirements of the EFAST algorithm. By consulting the crop model documentation and relevant literature, the initial values of the crop model parameters were set, and their variation ranges were specified to follow a uniform distribution within ±10%. The main model parameters and their value ranges are shown in Table 3.

**Table 3.** Main parameters and their value ranges for the WOFOST crop model

Parameter	Definition	Unit	Lower limit	Upper limit
TSUMEM	Growing degree days from sowing to emergence	°C·d	0	30
TBASEM	Emergence base temperature	°C	0	30
TSUM1	Growing degree days from emergence to flowering	°C·d	500	1,000
TSUM2	Growing degree days from flowering to maturity	°C·d	500	1,000
TDWI	Total initial dry matter	kg·ha^−1^	0.01	10
LAIEM	Leaf area index at emergence	ha·ha^−1^	0.0001	1
RGRLAI	Maximum growth rate of leaf area index	ha·ha^−1^	0.0001	1
SLATB	Specific leaf area	ha·kg^−1^	0	0.01
SPA	Specific pod area	ha·kg^−1^	0	0.01
SPAN	Leaf senescence coefficient	d	20	50
TBASE	Base temperature for leaf age	°C	−10	10
KDIFTB	Scattering extinction coefficient	-	0	1
EFFTB	Single leaf effective light energy use efficiency	kg·ha^−1^·h^−1^/(J·m^−2^·s^−1^)	0	1
AMAXTB	Maximum CO_2_ assimilation rate	kg·ha^−1^·h^−1^	0	60
TMPFTB	Calibration factor for maximum CO_2_ assimilation rate	-	0	1
CVL	Leaf assimilate conversion efficiency	kg·kg^−1^	0.2	1
CVO	Storage organ assimilate conversion efficiency	kg·kg^−1^	0.2	1
CVR	Root assimilate conversion efficiency	kg·kg^−1^	0.2	1
CVS	Stem assimilate conversion efficiency	kg·kg^−1^	0.2	1
RML	Maintenance respiration rate of leaves	kg·CH_2_O·kg^−1^·d^−1^	0	0.1
RMO	Maintenance respiration rate of storage organs	kg·CH_2_O·kg^−1^·d^−1^	0	0.1
RMR	Maintenance respiration rate of roots	kg·CH_2_O·kg^−1^·d^−1^	0	0.1
RMS	Maintenance respiration rate of stems	kg·CH_2_O·kg^−1^·d^−1^	0	0.1
FLTB	Leaf dry matter distribution coefficient	-	0	1
FOTB	Storage organ dry matter distribution coefficient	-	0	1
FSTB	Stem dry matter distribution coefficient	-	0	1

Among them, SLATB and KDIFTB analyze growth stages at 0.00 and 2.00, and EFFTB analyzes light energy utilization at temperatures between 0 and 40 °C. AMAXTB was analyzed for 3 growth stages: 0.00, 1.00, and 2.00. The TMPFTB was set at 8 temperature thresholds (0, 3, 10, 15, 20, 30, 35, and 40 °C) for the analysis of the calibration factor for the maximum CO₂ assimilation rate. FLTB, FOTB, and FSTB, 3 allocation coefficients, were analyzed for 8 growth stages (0.0, 0.3, 0.7, 1.0, 1.3, 1.5, 1.7, and 2.0).

The distribution coefficients (FLTB, FOTB, and FSTB) are relatively unique, requiring that the sum of the 3 coefficients be equal to 1 during the same growth period. This constraint makes it challenging to conduct parameter sensitivity analysis directly. Therefore, this study designed specific sensitivity analysis methods, and the steps were as follows:

1. Utilizing the SALib library in Python, the EFAST dataset DATA_Ori was generated on the basis of the crop parameters and their upper and lower limits.

2. Employing Simlab 2.2.1, constraints were imposed on the distribution coefficients via Eq. 7, and the dataset DATA_New was generated for the distribution coefficients.

$${\text{FLTB}}_{\mathrm{DVS}}+{\text{FSTB}}_{\mathrm{DVS}}+{\text{FOTB}}_{\mathrm{DVS}}=1$$ (7)

where FLTB, FSTB, and FOTB represent the distribution coefficients for leaves, stems, and siliques, respectively, and the sum of the 3 in the same growth period must equal 1. According to the constraint conditions, FLTB, FSTB, and FOTB should be constrained within the specified ranges on the basis of Eqs. 8 to 10:

$${\text{FLTB}}_{\mathrm{DVS}}\in \left[0,1\right]$$ (8)

$${\text{FSTB}}_{\mathrm{DVS}}\in \left[0,1-{\text{FLTB}}_{\mathrm{DVS}}\right]$$ (9)

$${\text{FOTB}}_{\mathrm{DVS}}=1-{\text{FLTB}}_{\mathrm{DVS}}-{\text{FSTB}}_{\mathrm{DVS}}$$ (10)

3. DATA_New was overwritten into DATA_Ori, and the sensitivity analysis was continued.

The above-ground biomass (TAGP) and total weight of storage organs (TWSO) in the WOFOST crop model can reflect the growth and yield formation status of rape. These indicators are closely related to the utilization efficiency of sunlight and CO_2_ by rape plants. Therefore, in this study, the TAGP and TWSO methods were used primarily as model output indicators for parameter sensitivity analysis.

## Results

### Parameter sensitivity analysis

**Fig. 6.**
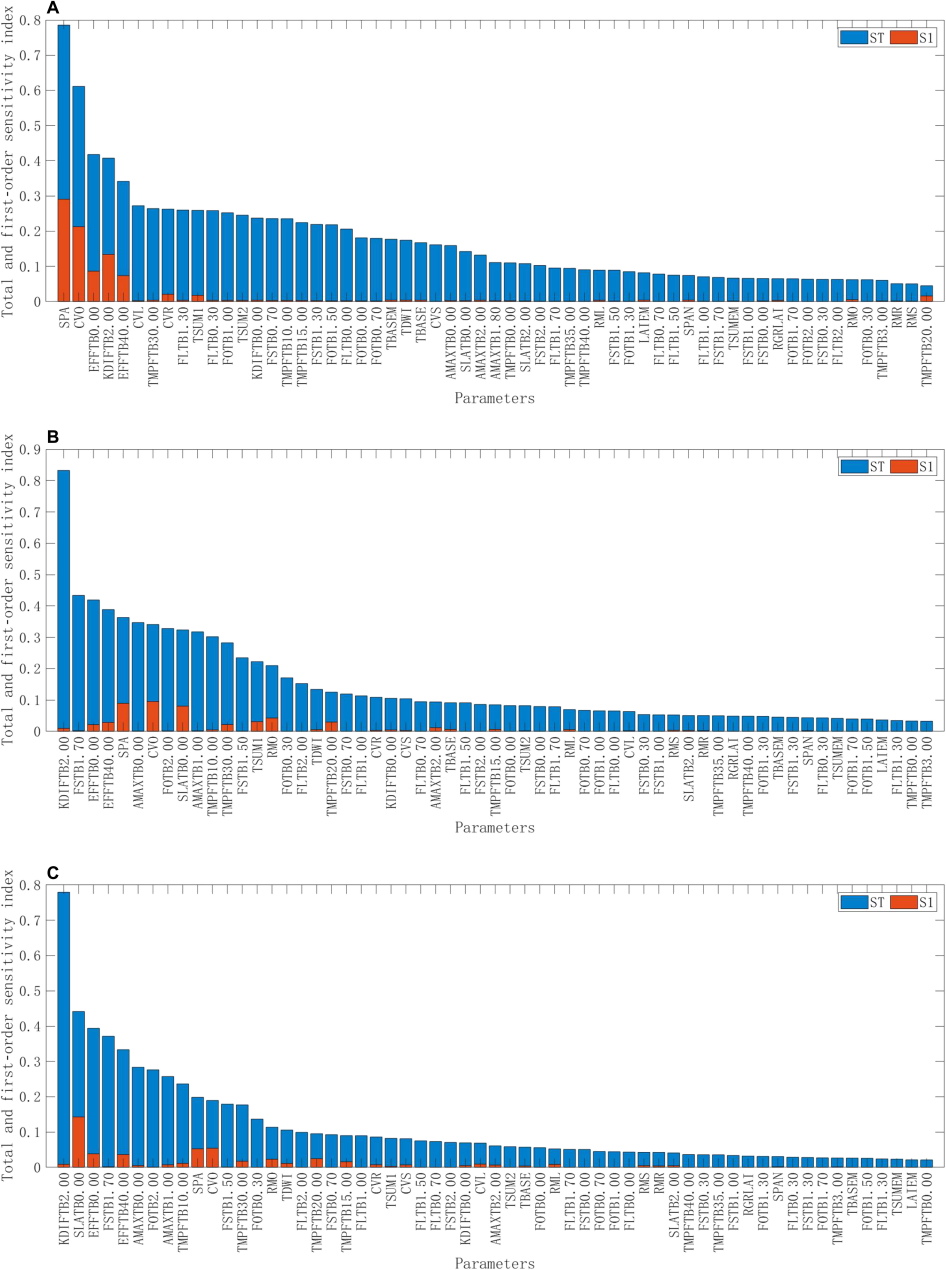
Sensitivity analysis results. (A) LAIMAX. (B) TWSO. (C) TAGP. Notes: S1 represents first-order sensitivity, which measures the contribution of individual parameters to the output, and ST represents global sensitivity, which describes the contribution of a parameter to the output after interacting with other parameters.

Figure 6 shows the sensitivity of various parameters in the WOFOST crop model to the maximum LAI (LAIMAX), TWSO, and aboveground biomass (TAGP). In the figure, S1 represents first-order sensitivity, which measures the contribution of individual parameters to the output, and ST represents global sensitivity, which describes the contribution of a parameter to the output after interacting with other parameters.

The sensitivity analysis results in Fig. 6 show that the SPA, storage organ assimilation efficiency (CVO), light use efficiency at 0 °C (EFFTB0.00), scattering extinction coefficient at growth stage 2 (KDIFTB2.00), and leaf assimilation efficiency (CVL) are highly sensitive to simulated rape growth under LAIMAX. KDIFTB2.00, the stem dry matter distribution coefficient at growth stage 1.7 (FSTB1.70), EFFTB0.00, the light use efficiency at 40 °C (EFFTB40.00), and the maximum CO_2_ assimilation rate at growth stage 0 (AMAXTB0.00) are highly sensitive to TWSO during simulated rape growth. KDIFTB2.00, FSTB1.70, EFFTB0.00, specific leaf area at growth stage 0 (SLATB0.00), and EFFTB40.00 exhibited high sensitivity to simulated rape TAGP.

Considering the comprehensive results of the parameter sensitivity analysis, the parameters with strong sensitivity to various indicators were KDIFTB2.00, EFFTB0.00, SPA, FSTB1.70, CVO, EFFTB40.00, and SLATB0.00. These parameters have a significant effect on the growth and yield of rape, making them suitable for calibrating crop model parameters. Furthermore, there was a strong correlation between parameters such as SPA, CVO, and CVL with the silique, demonstrating high levels of sensitivity and indicating the important role of the growth cycle of rape silique in the photosynthetic process. Compared with the results of a sensitivity analysis for cereal crops [27,41,42], parameters such as TSUM1, CVO, CVL, FOTB, KDIFTB, and EFFTB showed similar sensitivities, whereas higher levels were found for SPA in the sensitivity analysis of rape. This confirms that the parameters SPA and TPAI play crucial roles in achieving high-precision growth and yield simulations of rape. Therefore, new methods for rape crop model calibration are needed.

### Rape yield simulation

In this study, a single-point yield simulation of rape was conducted on the basis of the parameter sensitivity analysis results from the previous section via the WOFOST model. During the single-point yield simulation, it is necessary to calibrate the parameters of the crop model. The key parameters for crop model calibration are detailed in Table 4.

**Table 4.** Parameters for the rape single-point yield simulation based on the WOFOST model

Parameter	Unit	Mean	Minimum	Maximum
WAV	cm	42	25	55
TSUM1	°C·d	701	550	860
TSUM2	°C·d	737	650	850
TDWI	kg·ha^−1^	8.9	4.0	13.0
SPA	ha·kg^−1^	0.00065	0.00020	0.00090
SPAN	d	37.4	31.5	49.5
CVL	kg·kg^−1^	0.439	0.220	0.700
CVO	kg·kg^−1^	0.612	0.350	0.950
CVR	kg·kg^−1^	0.526	0.290	0.700
CVS	kg·kg^−1^	0.350	0.200	0.595
FLTB0.00	-	0.94	0.90	1.00
FLTB0.30	-	0.90	0.70	1.00
FLTB0.70	-	0.34	0.18	0.60
FLTB1.00	-	0.16	0.00	0.32
FLTB1.30	-	0.04	0.00	0.15
FLTB1.50	-	0.00	0.00	0.00
FLTB1.70	-	0.00	0.00	0.00
FLTB2.00	-	0.00	0.00	0.00
FOTB0.00	-	0.06	0.00	0.10
FOTB0.30	-	0.10	0.00	0.30
FOTB0.70	-	0.66	0.40	0.82
FOTB1.00	-	0.59	0.45	0.85
FOTB1.30	-	0.23	0.05	0.45
FOTB1.50	-	0.04	0.00	0.10
FOTB1.70	-	0.00	0.00	0.00
FOTB2.00	-	0.00	0.00	0.00
FSTB0.00	-	0.00	0.00	0.00
FSTB0.30	-	0.00	0.00	0.00
FSTB0.70	-	0.01	0.00	0.10
FSTB1.00	-	0.25	0.00	0.48
FSTB1.30	-	0.73	0.50	0.90
FSTB1.50	-	0.96	0.90	1.00
FSTB1.70	-	1.00	1.00	1.00
FSTB2.00	-	1.00	1.00	1.00

**Fig. 7.**
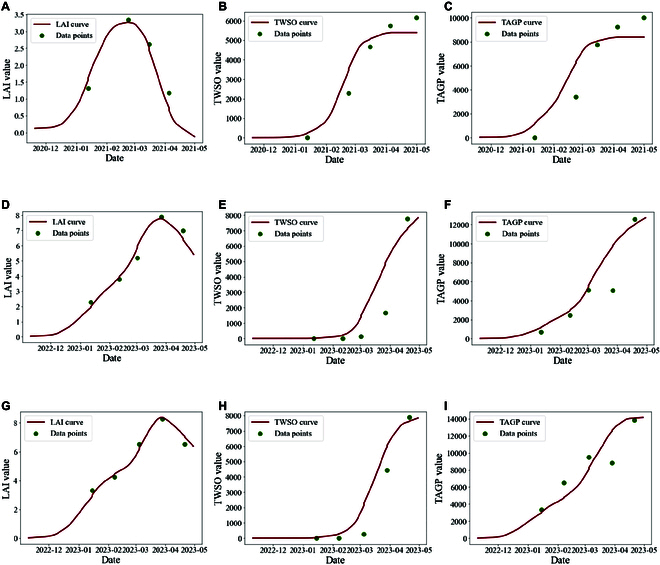
Temporal curves obtained via the LAI, TPAI-SPA, and TPAI-Curve calibration methods. While the green dots represent the measured data, the red curve represents the values simulated by the WOFOST crop model. (A) LAI. (B) TWSO. (C) TAGP. (D) TPAI-SPA LAI. (E) TPAI-SPA TWSO. (F) TPAI-SPA TAGP. (G) TPAI-Curve LAI. (H) TPAI-Curve TWSO. (I) TPAI-Curve for the TAGP.

On the basis of field-measured LAI and TPAI data, the temporal curves obtained via the LAI, TPAI-SPA, and TPAI-Curve calibration methods are shown in Fig. 7.

As shown in Fig. 7, the growth of rape was simulated via the LAI, TPAI-SPA, and TPAI-Curve methods using the WOFOST crop model. The LAI or TPAI values simulated via the model were calibrated to match the observed LAI or TPAI values. However, because the model that uses the LAI calibration method lacks dry matter accumulation simulations for photosynthesis in the rape silique after the flowering period, the yield indicators (TWSO and TAGP) failed to reach the observed values. Using the 2 new calibration methods (TPAI-SPA and TPAI-Curve), the simulated results were generally consistent with the observed values across most time points, with only the fourth simulation value slightly exceeding the observed value. This demonstrates the superiority of these 2 new methods for enhancing simulation accuracy.

**Fig. 8.**
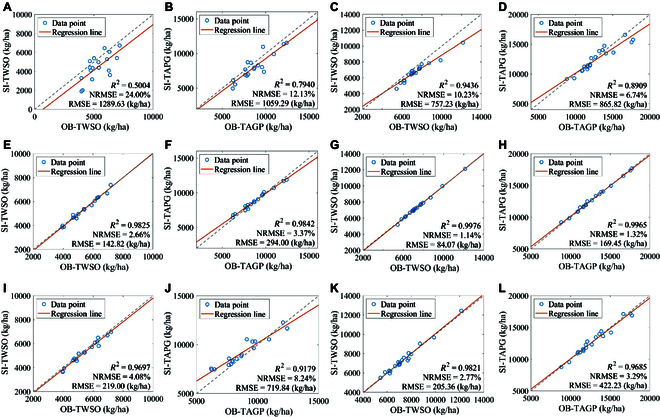
Modeling accuracy via the LAI, TPAI-SPA, and TPAI-Curve calibration methods. The green dots represent the measured data, and the red curve represents the values simulated by the WOFOST crop model. (A) 20/21 TWSO. (B) 20/21 TAGP. (C) 22/23 TWSO. (D) 22/23 TAGP. (E) 20/21 TPAI-SPA TWSO. (F) 20/21 TPAI-SPA TAGP. (G) 22/23 TPAI-SPA TWSO. (H) 22/23 TPAI-SPA TAGP. (I) 20/21 TPAI-Curve TWSO. (J) 20/21 TPAI-Curve TAGP. (K) 22/23 TPAI-Curve TWSO. (L) 22/23 TPAI-Curve TAGP.

**Fig. 9.**
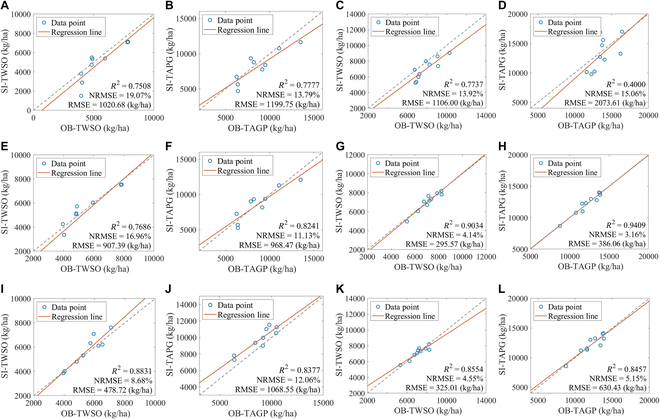
The validation accuracy was assessed via the LAI, TPAI-SPA, and TPAI-Curve calibration methods. The green dots represent the measured data, and the red curve represents the values simulated by the WOFOST crop model. (A) 20/21 TWSO. (B) 20/21 TAGP. (C) 22/23 TWSO. (D) 22/23 TAGP. (E) 20/21 TPAI-SPA TWSO. (F) 20/21 TPAI-SPA TAGP. (G) 22/23 TPAI-SPA TWSO. (H) 22/23 TPAI-SPA TAGP. (I) 20/21 TPAI-Curve TWSO. (J) 20/21 TPAI-Curve TAGP. (K) 22/23 TPAI-Curve TWSO. (L) 22/23 TPAI-Curve TAGP.

On the basis of field-measured LAI and TPAI data from rape fields in Hengyang city from 2020–2021 and 2022–2023, the LAI method, TPAI-SPA method, and TPAI-Curve method were employed to simulate the TAGP and TWSO yield indicators via the WOFOST crop model. The spatial variability of crop parameters in field crops is relatively small; therefore, we used a single set of crop parameters within one county. We input crop parameters obtained from calibration points, along with measured meteorological data, field management parameters, and other relevant data, into a crop model to simulate and validate yield indicators such as TWSO at the validation points. The measured data at the validation points were used to verify the simulation results at these points. The modeling results and validation accuracy are presented in Figs. 8 and 9, respectively. In this study, a ratio of 2:1 between the model calibration points and experimental validation points was maintained among the sample points in the study area to ensure the accuracy and reliability of the simulation results. Dispersed sampling was used as the field sampling method from 2020–2021, whereas from 2022–2023, dispersed sampling among counties and centralized sampling within counties were used.

As shown in Fig. 8A to D, the *R*^2^, RMSE, and NRMSE of the LAI method calibration model of TWSO were 0.7220, 1,023.43 kg/ha, and 17.12%, respectively. Similarly, for the TAGP, the values were 0.8427, 962.56 kg/ha, and 9.44%, respectively. Moreover, in the 2020–2021 experiments, the simulated values of TWSO and TAGP were underestimated by 16.53% and 7.42%, respectively. In the 2022–2023 experiments, the underestimation rates were 8.63% and 2.66%, respectively. Therefore, the crop model calibration method based on the LAI underestimates the simulated yield values. This finding indicates that under water stress conditions, the WOFOST crop model calibrated solely on the basis of LAI calibration has certain limitations in simulating rape yield.

In the experiments designed to calibrate the model via the TPAI-SPA and TPAI-Curve methods, which are based on the TPAI, the accuracies of both the TWSO and the TAGP were improved to a certain degree. As shown in Fig. 8E to H, when the TPAI-SPA method was used to calibrate the model, the *R*^2^ values of TWSO and TAGP increased by 37.13% and 17.53%, respectively, whereas the NRMSE decreased by 88.90% and 75.15%, respectively. Similarly, as shown in Fig. 8I to L, when the TPAI curve method was used to calibrate the model, the *R*^2^ values of TWSO and TAGP increased by 35.17% and 11.93%, respectively, whereas the NRMSE decreased by 79.99% and 38.90%, respectively. The precision of the 2 new methods based on the TPAI for calibrating the model was relatively high, and both exhibited varying degrees of improvement compared with the LAI method. These findings suggest that these 2 methods are suitable for calibrating the rape growth model.

The validation accuracies are shown in Fig. 9A to D, and the *R*^2^, RMSE, and NRMSE values of the model using the LAI method for TWSO were 0.7623, 1,063.34 kg/ha, and 16.50%, respectively. Similarly, for the TAGP, the values were 0.5889, 1,636.68 kg/ha, and 14.43%, respectively. In the 2020–2021 experiments, the simulated values of TWSO and TAGP were underestimated by 9.98% and 5.84%, respectively. In the 2022–2023 experiments, the underestimation rates were 11.35% and 3.75%, respectively. The validation experiment and the calibration experiment revealed similar underestimations. This demonstrates that it is difficult to simulate rape yield accurately when only crop models calibrated with LAI are used.

In the validation experiments conducted to calibrate the model via the TPAI-SPA and TPAI-Curve methods, which are based on the TPAI, the accuracies of both the TWSO and the TAGP improved to a certain degree. As shown in Fig. 9E to H, when the TPAI-SPA method was used for model calibration, the *R*^2^ values of TWSO and TAGP increased by 9.68% and 49.87%, respectively, whereas the NRMSE decreased by 36.04% and 50.47%, respectively. Similarly, as shown in Fig. 9I to L, when the TPAI-Curve method was used for model calibration, the *R*^2^ values of TWSO and TAGP increased by 14.04% and 42.60%, respectively, whereas the NRMSE decreased by 59.90% and 40.35%, respectively. The 2 new methods based on the TPAI demonstrated comparable accuracy to the calibration model in the validation experiments, and their performance was superior to that of the LAI method. These findings suggest that the TPAI-SPA and TPAI-Curve methods, which rely on the TPAI, are viable options for calibrating the rape growth model.

### TWSO in the research area

**Fig. 10.**
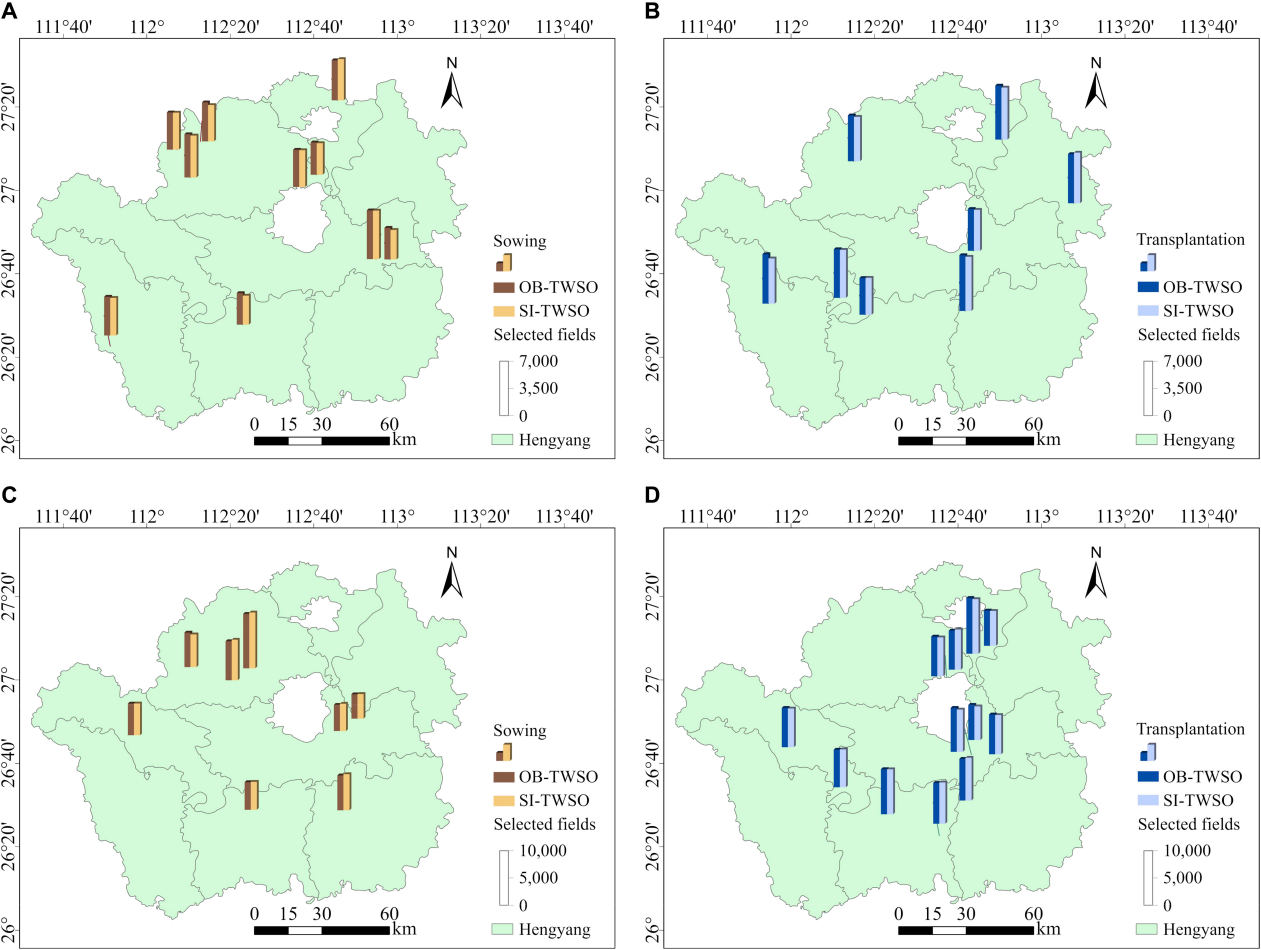
Simulation results of the TWSO of rape in the research area. Field sampling methods: 2020–2021, scattered sampling; 2022–2023, combination of intercounty scattered sampling and intracounty centralized sampling. (A) 2020–2021 sowing. (B) 2020–2021 transplantation. (C) 2022–2023 sowing. (D) 2022–2023 transplantation.

In addition, this study compared observed data and simulated yield data based on the TPAI for rape from 2020 to 2021 and from 2022 to 2023 and conducted yield distribution analysis at the city level, as shown in Fig. 10.

As shown in Fig. 10, the *R*^2^ values for the transplanting methods from 2020–2021 and 2022–2023 were 0.9287 and 0.9710, respectively, with corresponding NRMSE values of 4.06% and 2.31%, respectively. For the sowing methods, the *R*^2^ values for the same periods were 0.9667 and 0.9895, with NRMSE values of 4.03% and 3.30%, respectively. The spatial distribution maps of rape yield from 2020–2021 and 2022–2023 indicate that regardless of the planting method (transplantation or sowing) or the sampling method (scattered sampling or a combination of scattered and centralized sampling), the observed and simulated values of rape yield are highly consistent. The results demonstrate that the TPAI-based WOFOST crop model calibration methods proposed in this study exhibit high precision for yield prediction, further validating the feasibility of both the TPAI-SPA and TPAI-Curve calibration methods.

## Discussion

### Advantages of the TPAI in crop growth simulation and yield estimation

In crop growth models, the simulation of crop growth and yield is primarily based on the theory of crop photosynthesis [43]. For cereals such as wheat, corn, and rice, leaves are the main photosynthetic organs throughout the entire growth period. However, leaves are not the only organs in crops capable of performing photosynthesis. Non-foliar green organs such as pods, siliques, stems, and petioles also contribute to photosynthesis and ultimately to crop yield [31]. Rape exhibits a unique succession among photosynthetic organs and presents the most active non-foliar green organs. The non-foliar green organs, particularly siliques, play crucial roles in yield formation [44,45]. During the seedling to flowering stages of rape, leaves are the primary canopy component, performing most of the photosynthesis during the seedling stage. After flowering, siliques begin to grow, and their surface area rapidly increases. Currently, both leaves and siliques are components of the rape canopy and contribute to photosynthesis. During the silique development stage, as the siliques mature and the leaves degrade, rape relies primarily on photosynthesis of the silique wall for seed filling. At this time, the rape siliques become the primary component of the canopy. Throughout the growth periods of rape plants, the maximum area of silique walls was 1.54 times larger than the maximum leaf area. Furthermore, the net photosynthetic rate, transpiration rate, and light radiation intensity of the silique layer are greater than those of the leaf layer [17]. Additionally, research by Singal indicated that approximately 30% of rape yield originates from photosynthesis in leaves, whereas 70% originates from photosynthesis in silique walls [46]. Therefore, when developing a yield estimation system for rape, it is crucial to consider the characteristics of the rape plant and the mechanisms of yield formation.

Some scholars have improved crop models to achieve high-precision simulations of rape yield. Gilardelli et al. [47] conducted extensive modifications to the WOFOST crop model, achieving high-precision yield simulations of rape by focusing on crop detailed mechanistic supplements. However, in actual field experiments, it is still difficult to measure crucial parameters directly. Moreover, these improved models have reduced their applicability for simulating the yields of other crops. The TPAI-based method we propose does not involve modifications within the crop model itself. Instead, it substitutes the LAI externally with the TPAI, a parameter reflecting the photosynthesis of active non-leaf green organs in rape plants. After this improvement, the crop model remains applicable for simulating the yields of other crops. Moreover, different crop models vary significantly in their input requirements [23,48] and output objectives [49]. A direct comparison of their performance can indeed have poor comparability, and comparative studies of models will be conducted further in the future.

### Calibration methods for the WOFOST model based on the TPAI

This study proposes 2 calibration methods for the WOFOST model based on the TPAI. One is the TPAI-SPA calibration method, which is based on the TPAI and the specific photosynthetic area of siliques. The other method is the TPAI-Curve method, which is based on the TPAI and curve fitting. A comparison of the measured data with the simulation results revealed that both proposed WOFOST model calibration methods significantly improved the accuracy of the model in simulating rape yield. For the yield indicator TWSO, the TPAI-SPA and TPAI-Curve methods increased the estimation accuracy by 9.68% and 14.04%, respectively, compared with the traditional LAI method. This demonstrated the high accuracy of the proposed methods in simulating the accumulation of dry matter in rape storage organs. For the yield indicator TAGP, both the TPAI-SPA and TPAI-Curve methods achieved higher estimation accuracies than did the traditional LAI-based models, increasing the accuracy by 49.87% and 42.60%, respectively. These findings indicated that the proposed methods also exhibited high precision in simulating above-ground biomass for rape. When comparing the 2 proposed calibration methods, in the simulation of TWSO, the TPAI-Curve method demonstrated greater accuracy, further improving it by 3.98% compared with the TPAI-SPA method. Conversely, in the simulation of the TAGP, the TPAI-SPA method outperformed the TPAI-Curve method by 5.10%. Therefore, in experiments emphasizing high accuracy, a comprehensive approach involving both methods for simulating yield indicators might be considered. In summary, the 2 crop model calibration methods proposed in this study, the TPAI-SPA and the TPAI-Curve, exhibited high accuracy in simulating the growth and yield of rape in the study area. These methods, which employ different model calibration strategies, improve the accuracy of rape yield simulations to provide more reliable decision support for agricultural production.

Additionally, from the sensitivity analysis results, compared with cereals [27,41,42], both the TAGP and TWSO of rape are sensitive to the parameters TSUM1, CVO, CVL, FOTB, KDIFTB, and EFFTB. These findings indicate that the CO_2_ absorption rate during the seedling–flowering period, accumulated temperature sum, illumination during the silique–ripening period, rape light energy conversion efficiency, and nutrient allocation to stems and siliques all significantly affect yield formation. Notably, compared with cereals, the SPA parameter was more sensitive to the growth and yield formation processes of rape. This confirms the crucial role of SPA within the WOFOST crop model in simulating the TPAI, which plays a key role in achieving high-precision growth and yield simulations for rape.

In conclusion, the calibration of the TPAI based on the inclusion of the SAI, which is representative of general crop models such as WOFOST, significantly enhances the accuracy of rape yield simulations. This aligns with the agricultural mechanism in which the photosynthesis of siliques plays a crucial role in yield formation, as supported by the findings of Müller and Munné-Bosch [43] and others in related studies. Therefore, in the application of crop models for yield estimation, fully considering the impact of non-foliar green organs, such as siliques, on crop yield is essential to further improve the accuracy of crop yield estimation in crop modeling applications.

### Limitations and future improvements

Although this article has developed 2 new model calibration methods for rape that have shown high accuracy, this method is characterized by cumbersome data collection and processing. For example, during data collection, additional measurements of the diameter and length of the siliques and leaves are needed. During data processing, the discrete observation TPAI is used to establish regression curves and introduce them into the model, which increases the complexity of the experimental process.

In addition, this study did not include in-depth research on the simulation of regional rape yield estimation based on the WOFOST crop model. However, recently, researchers such as Huang et al. [19], Junior et al. [39], and Wu et al. [50] have explicitly highlighted the feasibility of using data assimilation techniques for regional crop yield estimation. As a result, the focus of future research will shift toward high-precision single-point simulations of regional rape using data assimilation techniques combined with satellite imagery. Specifically, the TPAI obtained from remote sensing inversion can be used as observation data through data assimilation techniques and compared with real-time TPAI data from the WOFOST crop model operation to dynamically calibrate the model state and simulate crop growth. The iterative optimization of the model can reflect real crop photosynthesis and yield accumulation status and better predict crop yield. This approach aims to increase the accuracy of regional rape yield estimation by integrating advanced technologies and addressing the spatial variability inherent in rape growth.

## Conclusion

To address the underestimation of rape yield estimates by traditional gramineous crop yield simulation methods based on crop models, this study utilized 2 years of field-measured data to conduct research on rape yield estimation for the southern Hunan region. This approach considers photosynthesis in the non-foliar green organs of rape and employs the WOFOST crop model. First, this study introduced the TPAI into the WOFOST crop model, which considers silique photosynthesis to improve the simulation accuracy of rape yield. Furthermore, 2 rape-specific parameter calibration methods based on the WOFOST crop model, namely, TPAI-SPA and TPAI-Curve, were proposed. Using the EFAST parameter sensitivity analysis method, this study identified parameters that significantly impact rape yield simulation, including TSUM1, CVO, CVL, and SPA, which theoretically verified the feasibility of TPAI calibration. Finally, the WOFOST crop model was used to simulate the measured rape yield data from 2020 to 2021 and from 2022 to 2023, and the TPAI-SPA and TPAI-Curve calibration methods were verified in the experiment. The research results indicate that, compared with traditional calibration methods of the WOFOST crop model, the *R*^2^ values of TWSO and TAGP improved by 9.68% and 49.87%, respectively, when the TPAI-SPA method was used and by 14.04% and 42.60%, respectively, when the TPAI-Curve method was used. These findings demonstrate the significant effectiveness of the TPAI-SPA and TPAI-Curve methods in improving the accuracy of rape yield estimation via the WOFOST crop model.

## Data Availability

The code and training script of TPAI-SPA and TPAI-Curve has been hosted to GitHub and is available at https://github.com/rsw1998/TPAI-SPA-Curve-for-WOFOST.
